# Three-Dimensional Assessment and Comparison of the Maxillary Tuberosity Between Skeletal and Dental Class I and Class II Adults in Maxillary Third Molar Agenesis Using Cone Beam Computed Tomography: A Descriptive Cross-Sectional Human Study

**DOI:** 10.7759/cureus.42232

**Published:** 2023-07-21

**Authors:** Nachu Lakshmi Manojna, Ghanta Sunil, Kotha Ramya, Inuganti Ranganayakulu, RSVM Raghu Ram

**Affiliations:** 1 Orthodontics and Dentofacial Orthopedics, Vishnu Dental College, Bhimavaram, IND; 2 Orthodontics and Dentofacial Orthopedics, GSL Dental College & Hospital, Rajahmundry, IND; 3 Oral Medicine and Radiology, GSL Dental College & Hospital, Rajahmundry, IND

**Keywords:** malocclusion, maxillary third molar agenesis, cone beam computed tomography, anatomical dimensions, maxillary tuberosity

## Abstract

Aim and objective: The objective of this study was to assess and compare the dimensions (width (W), height (H), and length (L)) of the tuberosity distal to maxillary permanent second molar in individuals with skeletal and dental Class I and Class II malocclusions who had maxillary third molar agenesis.

Methodology: Cone beam computed tomography (CBCT) was used to measure the left (L) and right (R) anatomical tuberosity dimensions in three dimensions using the WillMaster software (HDX WILL Corporation, Korea). The measurements were compared between Class I (n = 35) and Class II (n = 35) normo-hypodivergent adult subjects. The dimensions were measured at regular 2 mm intervals from the cementoenamel junction (CEJ) and distovestibular root of the maxillary second molar in terms of the width (e.g., W1, W2, and W3), height (e.g., H1, H2, and H3), length (e.g., L1, L2, and L3) to the posterior limit of the tuberosity. Statistical analysis included descriptive statistics, Mann-Whitney U tests, and intraclass correlation coefficient tests.

Results: The width of the tuberosity at LW0, LW1, and LW2 was significantly higher in Class I compared to that in Class II. The right tuberosity in Class II showed significantly higher values in height at all reference points. The right tuberosity at RL0 and RL1 exhibited significantly higher values in the length of the Class II group compared to the Class I group.

Conclusions: The dimensions of the maxillary tuberosity (width, height, and length) varied between the Class I and Class II groups. Wider maxillary tuberosities were observed in the Class I group, while the Class II group had greater height and length dimensions of the tuberosity.

## Introduction

Advancements in orthodontic treatment have led to a shift from extraction cases to non-extraction procedures and from borderline surgical cases to nonsurgical treatments. Procedures, such as maxillary arch distalization, could only be performed when adequate space was available in the tuberosity area [1-3]. Nowadays, the congenital absence of maxillary third molars is frequently observed as an evolutionary process [4, 5]. The presence or absence of maxillary third molars significantly influences the dimensions of the tuberosity. In addition, the dimensions of the tuberosity area can be altered by the type of occlusal relationship. A thorough understanding of maxillary tuberosity, including its anatomy, dimensions, bone type, and surrounding structures, is essential, especially when performing maxillary teeth distalization in orthodontic therapy.

The maxillary tuberosity is a rounded projection of a compact bone that extends posteriorly from the alveolar crest, continuing the structure of the maxillary bone. It is mesially bounded by the last erupted molar and maxillary sinus, while distally it is bounded by the pyramidal process of the palatine bone and pterygopalatine fissure [6]. During development, the tuberosity initially consists of fine cancellous, non-lamellar bone tissue, which later becomes compacted into lamellar bone [7]. The dimensions of the tuberosity, including width, length, and height, vary with age.

The bony architecture of the tuberosity plays a significant role in achieving successful arch distalization with appropriate treatment mechanics [1]. The anatomy of the tuberosity, in all three dimensions, can affect arch distalization, and its unnoticed variations may lead to external root resorption, dehiscence, fenestrations, and tooth mobility [8]. Therefore, it is crucial to assess the clinical and anatomical limitations before initiating treatment. Traditional two-dimensional (2D) radiography has limitations, including magnification and superimposition of structures, which affect accurate diagnosis [9,10]. Although three-dimensional (3D) imaging systems, such as computed tomography (CT) scans, provide detailed information, they have drawbacks, such as high radiation exposure, limited spatial resolution of the teeth, and the need for image reconstruction into multiple thin slices [8,11]. By contrast, cone beam CT (CBCT) scans offer innovative imaging of the maxillomandibular regions, providing true-to-scale 3D voxel images in all three planes (sagittal, coronal, and transverse) [11-14] with 1:1 measurement and without superimposition of structures.

Only a limited number of studies have investigated the 2D aspects of the maxillary tuberosity region, making it less reliable for achieving optimal treatment outcomes. Previous studies have described the morphological types of the maxillary tuberosity based on age. Type 1, observed in younger patients, exhibits small width, large height, and short length. Type 2, found in middle-aged patients, shows larger width, lesser height, and average length. Type 3, seen in elderly patients, demonstrates average width, very small height, and large length [15]. However, there is a lack of anatomical studies assessing the 3D aspects of the maxillary tuberosity, particularly in the cases of maxillary third molar agenesis, and its variations between skeletal and dental Class I and Class II malocclusions. Therefore, it is necessary to investigate the dimensions of the maxillary tuberosity in skeletal and dental Class I and Class II adults with maxillary third molar agenesis. This study aimed to assess and compare the dimensional changes (width, height, and length) of the maxillary tuberosity distal to the maxillary permanent second molar (posterior bone limit) in adult subjects with maxillary third molar agenesis and normo-hypodivergent skeletal and dental Class I and Class II malocclusions using CBCT imaging.

## Materials and methods

The study protocol was approved by the GSL Dental College and Hospital Ethics Committee for Human Research (GSLDC/IEC/2019/018). All patients who required orthodontic treatment and reported to the Department of Orthodontics, GSL Dental College and Hospital, Andhra Pradesh, India, between August 2020 and January 2023, were included in the study. Detailed information about the study was provided to all patients, and informed consent was obtained for their participation.

A descriptive cross-sectional human study was conducted in accordance with the Strengthening the Reporting of Observational Studies in Epidemiology (STROBE) statement and complied with the guidelines outlined in the Declaration of Helsinki for research involving human subjects. The sample size for the quantitative variables was calculated using G*Power 3.1.9.2 software (IBM SPSS, Armonk, NY, USA), considering an effect size of 0.8 (Cohen's large effect size), α error probability of 5%, power of 90%, and an allocation ratio of 1:1 based on a pilot study. The calculated sample size was 68, with 34 subjects in each group (skeletal and dental - Class I and Class II) based on the inclusion criteria, which was rounded off to 35 in each group, resulting in a total study size of 70. Both groups comprised 24 females and 11 males, with a mean age of 39.7 ± 8.4 years. The inclusion criteria were as follows: (1) age over 18 years; (2) Point A (A) - Nasion (N) - Point B (B) [ANB] angle: Class I (ANB ± 2˚) and Class II (ANB ≥ 4˚); (3) normo-hypodivergent facial profiles Sella (S) - Nasion (N) (SN) - Mandibular Plane (MP) [SN-MP] angle ≤ 32˚; (4) Class I malocclusion; (5) Class II or bilateral end-on malocclusion; (6) bilateral agenesis of maxillary third molars; and (7) a full complement of permanent dentition, excluding third molars. The exclusion criteria were as follows: (1) facial asymmetry; (2) transverse/vertical skeletal or dental discrepancies; (3) periodontal diseases, endodontically treated permanent maxillary molars, prosthodontic rehabilitation of maxillary molars; and (4) previous orthodontic treatment.

CBCT scans of the patients were obtained using the DENTRI machine (HDX WILL Corporation, Korea), with a single 360˚ rotation, employing the following operational settings: tube voltage 85 kvp, tube current 8 mA, scan time 50 seconds, slice thickness 0.20 mm, and field of view 114 mm x 79 mm. The resulting images were saved as Digital Imaging and Communications in Medicine (DICOM) files, which were then reconstructed into 3D images as multiplanar reformatted images using the OnDemand 3D software (version 2016.12, Cybermed, Korea). The scanned images were imported into cephalometric analysis software (WillCeph digital imaging software, version 1.0.0; HDX Will Corp, Korea) for conventional lateral cephalometric analysis. Landmarks were identified, and planes were drawn on the CBCT-synthesized 2D cephalogram [16] to diagnose the skeletal jaw bases and confirm the growth pattern. The 3D images derived from the CBCT scans were viewed in the sagittal cross-sectional plane to establish the reference plane (maxillary occlusal) for measuring the maxillary tuberosity. The maxillary occlusal plane was defined as a line connecting the incisal edge of the maxillary central incisors to the buccal cusp tips of the first and second maxillary molars in the sagittal axis (lateral cephalogram view). Once the maxillary occlusal plane was established, it was reoriented and fixed onto the sectional views at the maxillary first and second molars, where the maximum length of the tuberosity was visualized. The frame of the slide section was fixed to measure the length and height of the maxillary tuberosity. With the maxillary occlusal plane as a reference, two new lines were drawn: a perpendicular line from the most prominent distal surface of the maxillary second molar and a parallel line from the cementoenamel junction (CEJ) of the maxillary second molar, set as the zero plane. To fix the axial cross-section for measuring the width of the tuberosity, the sagittal cross-section (coronal view) was positioned so that the buccal and palatal cortical plates of the maxillary tuberosity were parallel to a section passing through the center of the tuberosity. Subsequently, the axial cross-section frame was fixed at the maximum width of the bucco-palatal tuberosity area at the CEJ of the maxillary second molar. The width, length, and height of the maxillary tuberosity were measured on the right (R) and left (L) quadrants using the OnDemand 3D software.

**Figure 1:**
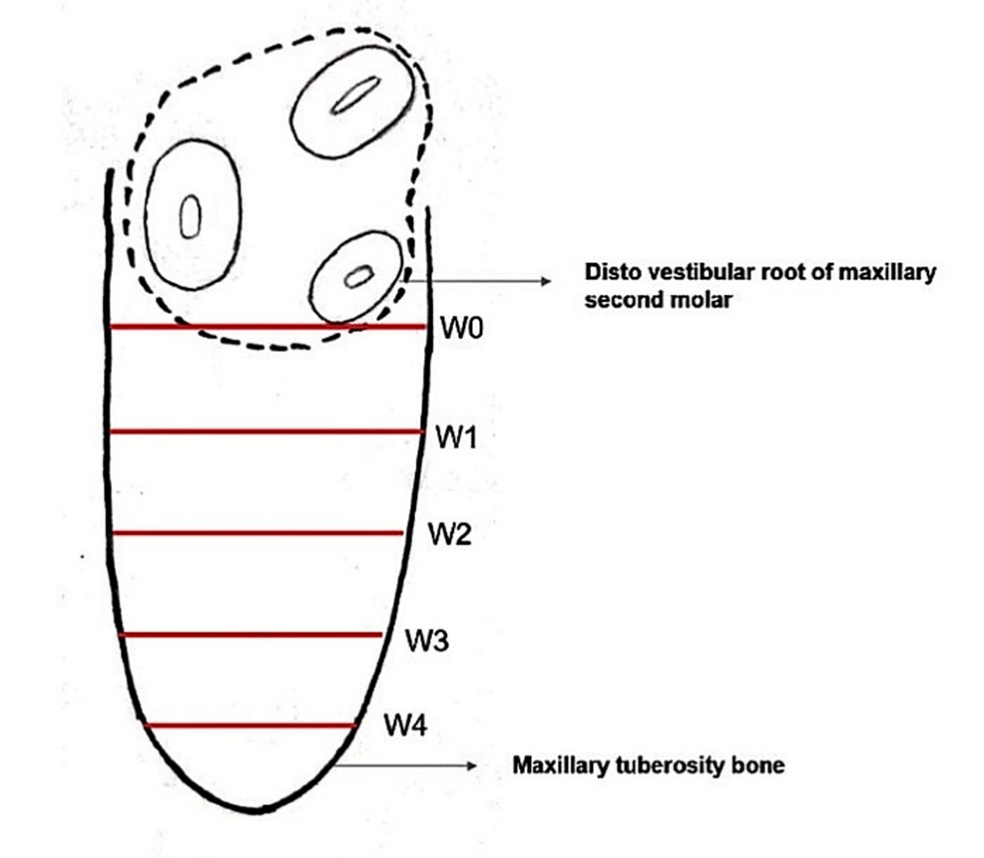
Diagrammatic depiction of the width of the maxillary tuberosity. W0: Initial line parallel to the distovestibular root surface. W1, W2, W3, W4: Consecutive linear distances (parallel to W0) measured at 2 mm intervals up to the posterior anatomical limit of the maxillary tuberosity.

The width (W) of the tuberosity was measured as the maximum vestibulo-lingual transverse distance from the most distal point of the distovestibular root of the maxillary second molar on the axial section. An initial line (W0) was made parallel to the distovestibular root surface, and consecutive linear distances (parallel to W0) were measured at 2 mm intervals up to the posterior anatomical limit of the maxillary tuberosity (Figure 1).

**Figure 2:**
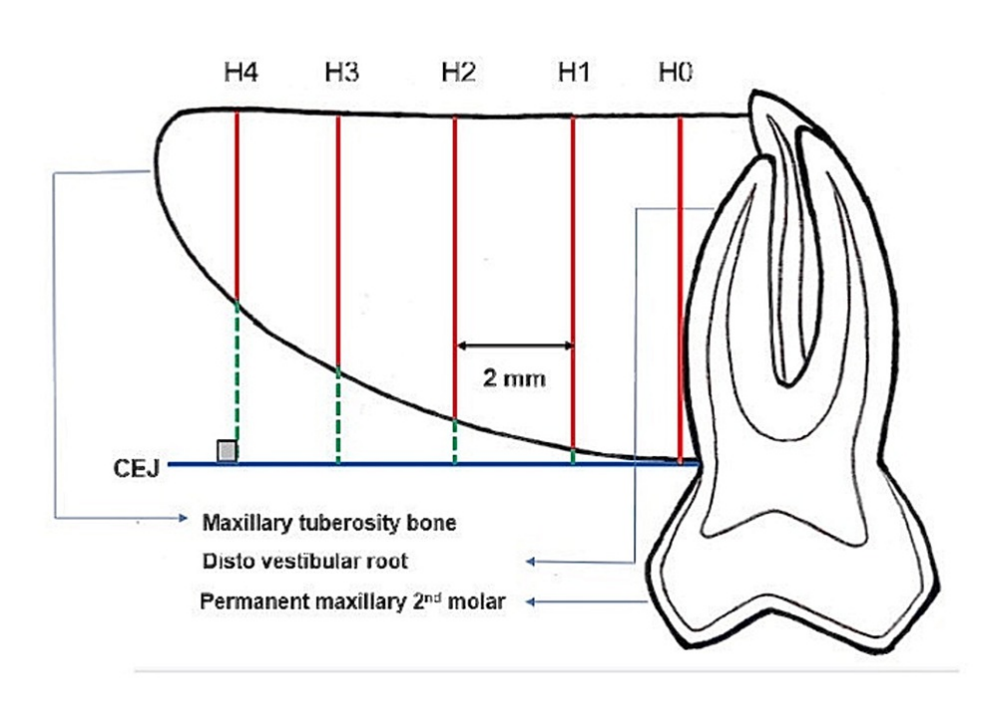
Diagrammatic depiction of the height of the maxillary tuberosity. H0: Initial line parallel to the distovestibular root surface of the upper second molar. H0, H1, H2, H3, H4: Consecutive perpendicular lines (parallel to H0) measured at 2mm intervals up to the posterior anatomical limit of the maxillary tuberosity.

The height (H) of the tuberosity was measured as the maximum vertical line, representing the occlusal-apical distance from the CEJ to the most apical point of the basal bone (tangent line to the maxillary antrum) of the tuberosity on a sagittal section. An initial line (H0), parallel to the distovestibular root surface of the upper second molar, and consecutive perpendicular lines (parallel to H0) were measured at 2 mm intervals up to the posterior anatomical limit of the maxillary tuberosity (Figure 2).

**Figure 3:**
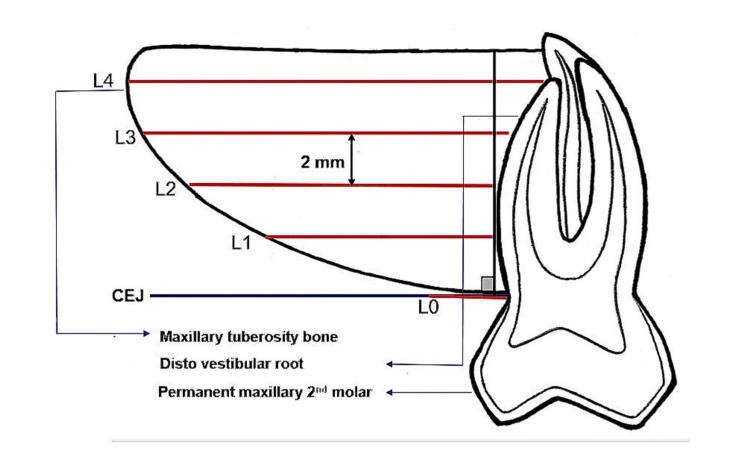
Diagrammatic depiction of the length of the maxillary tuberosity. L0: Initial line measured from the cemento-enamel junction of the second molar, parallel to the occlusal plane and perpendicular to the distovestibular root surfaces. L1, L2, L3, L4: Consecutive linear distances at 2 mm intervals (parallel to L0) measured up to the posterior anatomical limit of the maxillary tuberosity.

The length (L) of the tuberosity was measured as the anteroposterior distance from the most distal point of the vestibulo-distal root of the upper second molar to the most posterior point of the tuberosity on the sagittal view. An initial line (L0) was measured from the CEJ of the second molar, parallel to the occlusal plane and perpendicular to the distovestibular root surfaces. Consecutive linear distances at 2 mm intervals (parallel to L0) were measured as L1, L2, and so on, up to the posterior anatomical limit of the maxillary tuberosity (Figure 3).

All measurements were carried out by a single examiner, and after two weeks, 15 randomly selected CBCT scans were measured by the same examiner to assess intra-examiner reliability. The collected data were statistically analyzed using IBM SPSS Statistics for Windows, Version 20 (Released 2012; IBM Corp., Armonk, New York, United States). Descriptive statistics, Mann-Whitney U tests, and intraclass correlation coefficient tests were employed to analyze the data. The statistical significance level was set at p ≤ 0.05.

## Results

Table 1 shows the comparison of cephalometric indicators in degrees between the skeletal and dental Class I and Class II groups. The Class II group exhibited a significantly higher mean Sella-Nasion-Point A (SNA) value (84.09 ± 0.91 degrees) compared to the Class I group (81.94 ± 0.87 degrees) (p = 0.001*). There was no significant difference in the Sella-Nasion-Point B (SNB) values between the groups. However, the Point A-Nasion-Point B (ANB) values were significantly greater in the Class II group (4.71 ± 0.66 degrees) than in the Class I group (2.43 ± 0.5 degrees) (p < 0.0001). The mandibular plane angle (MPA) values showed no significant difference between the groups. These findings highlight the differences in cephalometric measurements between the skeletal and dental Class I and Class II groups.

**Table 1:** Comparison of cephalometric indicators in degrees between the Class I and Class II groups. SD: standard deviation; IQR: interquartile range; Mann-Whitney U test; p ≤ 0.05 considered statistically significant; * denotes statistical significance SNA: Sella-Nasion-Point A; SNB: Sella-Nasion-Point B; ANB: Point A-Nasion-Point B; MPA: mandibular plane angle

Variable	Class I		Class II		MannWhitney U	p-value
	Mean ± SD	Median (IQR)	Mean ± SD	Median (IQR)		
SNA	81.94 ± 0.87	82 (2)	84.09 ± 0.91	84 (2)	64.5	0.001*
SNB	79.4 ± 0.881	79 (1)	79.09 ± 0.81	79 (1)	501	0.164
ANB	2.43 ± 0.5	2 (1)	4.71 ± 0.66	5 (1)	0.00	0.0001*
MPA	30.4 ± 1.39	31 (2)	30.49 ± 1.42	31 (2)	5.88	0.767

The parameters of width, height, and length were compared separately for the left and right sides between the groups. The intraclass correlation coefficient values ranged from 0.97 to 1, indicating good intra-observer reliability.

Table 2 displays the comparison of the width of the right and left maxillary tuberosity in millimeters between the Class I and Class II subjects at different reference points. For the right width (RW) measurements, no statistically significant differences were found between the skeletal and dental Class I and Class II groups at reference points RW0, RW1, and RW2 (p > 0.05). However, for the left width (LW) measurements, significant differences were observed between the skeletal and dental Class I and Class II groups at reference points LW0, LW1, and LW2 (p = 0.001). The Class I group had higher mean widths compared to the Class II group at these reference points. These findings indicate that there were variations in the width of the maxillary tuberosity between the skeletal and dental Class I and Class II subjects, particularly on the left side.

**Table 2:** Comparison of the width of the right and left maxillary tuberosity in millimeters between Class I and Class II subjects at different reference points. SD: standard deviation; IQR: interquartile range; Mann-Whitney U test; p ≤ 0.05 considered statistically significant; * denotes statistical significance RW: right width of the maxillary tuberosity; LW: left width of the maxillary tuberosity

Variable	Class I		Class II		Mann-Whitney U	p-value
	Mean ± SD	Median (IQR)	Mean ± SD	Median (IQR)		
RW0	10.55 ± 3.37	11.81 (3.54)	10.78 ± 1.32	10.6 (1.02)	522.5	0.210
RW1	8.37 ± 4.54	9.65 (5.89)	8.68 ± 2.15	9.21 (3.85)	549	0.456
RW2	5.19 ± 5.03	4.7 (10.19)	4.18 ± 3.46	4.3 (6.95)	546	0.423
LW0	12.09 ± 2.77	12.89 (2.65)	10.68 ± 1.69	10.89 (2.34)	327	0.001*
LW1	10.61 ± 2.58	9.9 (4.28)	7.37 ± 3.86	7.85 (6.46)	317	0.001*
LW2	7.28 ± 4.2	7.86 (5.13)	3.84 ± 3.71	4.27 (7.39)	341.5	0.001*

In Table 3, the height of the right and left maxillary tuberosity in millimeters between the skeletal and dental Class I and Class II subjects at different reference points were compared. Significant differences were observed in the right height (RH) measurements between the skeletal and dental Class I and Class II groups at reference points RH0, RH1, RH2, and RH3 (p < 0.05). The Class II group exhibited higher mean heights compared to the Class I group at these reference points. However, no statistically significant differences were found in the left height (LH) measurements between the two groups at reference points LH0, LH1, LH2, and LH3 (p > 0.05). These findings indicate variations in the height of the maxillary tuberosity between the skeletal and dental Class I and Class II subjects, primarily on the right side.

**Table 3:** Comparison of the height of the right and left maxillary tuberosity in millimeters between Class I and Class II subjects at different reference points. SD: standard deviation; IQR: interquartile range; Mann-Whitney U test; p ≤ 0.05 considered statistically significant; * denotes statistical significance RH: right height of the maxillary tuberosity; LH: left height of the maxillary tuberosity

Variable	Class I		Class II		Mann-Whitney U	p-value
	Mean ± SD	Median (IQR)	Mean ± SD	Median (IQR)		
RH0	9.96 ± 1.76	10.18 (1.19)	11.14 ± 3.31	12.48 (7.1)	398	0.01*
RH1	7.95 ± 3.95	8.5 (2.85)	11.22 ± 2.93	11.96 (4.43)	309.5	0.001*
RH2	4.67 ± 4.74	5.14 (9.14)	9.26 ± 3.36	8.41 (5.01)	346.5	0.002*
RH3	2.83 ± 3.83	0 (7.18)	5.48 ± 3.76	5.63 (3.92)	408	0.012*
LH0	10.71 ± 1.7	11.07 (1.52)	10.45 ± 2.96	11.01 (4.1)	611	0.986
LH1	9.52 ± 3.65	10.56 (1.82)	10.37 ± 3.56	11.39 (5.87)	458	0.07
LH2	5.84 ± 5.03	7.81 (10.52)	8.31 ± 3.72	9.75 (4.59)	465	0.081
LH3	4.63 ± 4.21	5.28 (8.25)	3.73 ± 3.63	3.89 (6.43)	534	0.341

In Table 4, the length of the right and left maxillary tuberosity in millimeters between the skeletal and dental Class I and Class II subjects at different reference points were compared. Significant differences were observed in the right length (RL) measurements between the skeletal and dental Class I and Class II groups at reference points RL0 and RL1 (p < 0.05). The Class II group exhibited higher mean lengths compared to the Class I group at these reference points. However, no statistically significant differences were found in the left length (LL) measurements between the two groups at any of the reference points (p > 0.05). These findings indicate variations in the length of the maxillary tuberosity between the skeletal and dental Class I and Class II subjects, primarily on the right side.

**Table 4:** Comparison of the length of the right and left maxillary tuberosity in millimeters between Class I and Class II subjects at different reference points. SD: standard deviation; IQR: interquartile range; Mann-Whitney U test; p ≤ 0.05 considered statistically significant; * denotes statistical significance RL: right length of the maxillary tuberosity; LW: left length of the maxillary tuberosity

Variable	Class I		Class II		Mann-Whitney U	p-value
	Mean ± SD	Median (IQR)	Mean ± SD	Median (IQR)		
RL0	8.79 ± 2.7	9.33 (4.3)	10.38 ± 2.1	9.73 (3.6)	395	0.011*
RL1	8.73 ± 2.64	9.02 (3.87)	10.16 ± 3.75	10.04 (2.97)	383	0.007*
RL2	8.47 ± 2.72	8.42 (3.44)	8.5 ± 4.94	10.39 (4.08)	467	0.087
RL3	4.94 ± 4.8	7.15 (9.07)	7.05 ± 5.5	8.97 (11.62)	455	0.056
LL0	10.43 ± 2.17	11.2 (4.48)	11.39 ± 1.92	11.35 (2.4)	485.5	0.136
LL1	10.22 ± 1.95	10.98 (3.37)	8.6 ± 4.8	10.37 (5.03)	529	0.326
LL2	9.83 ± 2.49	11.03 (3.55)	8.24 ± 4.88	9.82 (6.4)	531	0.338
LL3	6.76 ± 4.91	8.12 (10.76)	6.05 ± 5.24	8.38 (10.28)	586	0.75

## Discussion

Maxillary arch distalization was performed as a camouflage treatment in orthodontics for borderline extraction and surgical cases. The tuberosity was bounded mesially by the last erupted molar and maxillary sinus and distally by the pyramidal process of the palatine bone and pterygopalatine fissure. The bony architecture of the tuberosity was favorable for the distalization technique when performed carefully. Agenesis of the maxillary third molars was commonly observed as a result of tooth evolution [4,5]. The size and shape of the tuberosity could be influenced by the absence of the maxillary third molar. Therefore, there was a need to evaluate the anatomical dimensions posterior to the maxillary second molar in cases of third molar agenesis, particularly in normo-hypodivergent skeletal Class I and Class II adult subjects who required molar distalization.

In the present study, CBCT was utilized to overcome the limitations of routine radiographic views. CBCT provided true-to-scale 3D images with smaller voxel sizes, allowing for accurate measurements without distortion or superimposition of structures [11,13]. To eliminate the effects of growth, only subjects aged 18 and above were included in the study, as suggested by previous research [6,17].

Manzanera et al. conducted a study in which they measured the dimensions (width, height, and length) of the maxillary tuberosity distal to the maxillary second molar at various irregular intervals. However, they did not discuss the reason why they measured at various intervals in all three dimensions of planes. The widths of the tuberosity were measured at intervals of 3 mm, 4.5 mm, and 9 mm, resulting in values of 12.24 mm, 11.22 mm, and 9.10 mm, respectively. Likewise, the heights and lengths were measured at distances of 3 mm, 6 mm, and 9 mm, showing values of 10.06 mm, 10.46 mm, and 11.91 mm for height and 9.42 mm, 11.23 mm, and 11.25 mm for length, respectively [15].

A uniform distance of 2 mm was taken as a standard interval in the current study to ensure precise anatomical measurements of width, height, and length. Statistical significance was observed for all dimensions at almost all regular intervals. The width of the tuberosity was measured on both sides, with measurements taken every 2 mm interval. The study found that the widths of the tuberosity (transverse distances) from LW0 to LW2 showed a progressive reduction from the maxillary second molars to the posterior limit of the tuberosity. This tapered anatomy of the left maxillary tuberosity was statistically more pronounced in the Class II group (10.68 mm, 7.37 mm, and 3.84 mm) compared to the Class I group (12.09 mm, 10.61 mm, and 7.28 mm). On the right side, the tapered anatomy in the transverse dimension (width) was similar in both Class I and Class II groups (Table 2). The increase in tuberosity width might be influenced by factors, such as the side of mastication, type of malocclusion, and associated masticatory forces.

The height of the right tuberosity showed a significant variation in mean heights at all reference points from H0 to H3 in the Class II group (11.14 mm to 9.26 mm) compared to the Class I group (9.96 mm to 4.67 mm). It was observed that the height of the right maxillary tuberosity decreased anteroposteriorly, and the heights in the Class II group were comparatively larger than those in the Class I group. The height of the left tuberosity also decreased anteroposteriorly, with no statistical significance between the groups (Table 3). The height of the tuberosity was influenced by the extension of the maxillary sinus [6]. In the present study, the sinus was slightly higher in the Class II group, allowing for increased tuberosity height.

In terms of length, significant differences were found in the right tuberosity at L0 and L1, with the Class II group showing higher mean lengths (10.38 mm and 10.16 mm) compared to the Class I group (8.79 mm and 8.73 mm). However, both groups maintained a relatively stable length from L0 to L2/L3, followed by a sudden drop in length, giving the tuberosity an upward curved surface (Table 4).

The results obtained from the present study are relevant to the use of maxillary arch distalization in Class I and Class II adults with maxillary third molar agenesis, considering the dimensions of the tuberosity in all three dimensions. However, the study had limitations, including an uneven distribution of gender within each group for statistical comparison. Further studies are needed to explore the influence of various growth patterns on tuberosity dimensions.

## Conclusions

Skeletal and dental Class I adults with maxillary third molar agenesis exhibited a wider width of the tuberosity up to 2 mm distal to the maxillary second molars. The width of the left tuberosity was wider in Class I adults compared to Class II adults. In addition, Class II adults showed greater heights on the right tuberosity up to 4 mm from the distal surface of the maxillary second molars in cases of maxillary third molar agenesis. The right tuberosity from the cementoenamel junction of the maxillary second molar up to 4 mm also demonstrated increased length in Class II adults compared to the Class I group in cases of maxillary third molar agenesis.
